# Diagnostic and prognostic potential of the microbiome in ovarian cancer treatment response

**DOI:** 10.1038/s41598-023-27555-x

**Published:** 2023-01-13

**Authors:** Abigail E. Asangba, Jun Chen, Krista M. Goergen, Melissa C. Larson, Ann L. Oberg, Jvan Casarin, Francesco Multinu, Scott H. Kaufmann, Andrea Mariani, Nicholas Chia, Marina R. S. Walther-Antonio

**Affiliations:** 1 Department of Surgery, Mayo Clinic, Rochester, MN USA; 2 Microbiome Program, Center for Individualized Medicine, Mayo Clinic, Rochester, MN USA; 3 Department of Quantitative Health Sciences, Mayo Clinic, Rochester, MN USA; 4 Department of Obstetrics and Gynecology, Mayo Clinic, Rochester, MN USA; 5 IEO, European Institute of Oncology IRCCS, Milan, Italy; 6 Department of Oncology, Mayo Clinic, Rochester, MN USA

**Keywords:** Cancer, Computational biology and bioinformatics, Microbiology, Diseases

## Abstract

Ovarian cancer (OC) is the second most common gynecological malignancy and the fifth leading cause of death due to cancer in women in the United States mainly due to the late-stage diagnosis of this cancer. It is, therefore, critical to identify potential indicators to aid in early detection and diagnosis of this disease. We investigated the microbiome associated with OC and its potential role in detection, progression as well as prognosis of the disease. We identified a distinct OC microbiome with general enrichment of several microbial taxa, including *Dialister*, *Corynebacterium*, *Prevotella*, and *Peptoniphilus* in the OC cohort in all body sites excluding stool and omentum which were not sampled from the benign cohort. These taxa were, however, depleted in the advanced-stage and high-grade OC patients compared to early-stage and low-grade OC patients suggestive of decrease accumulation in advanced disease and could serve as potential indicators for early detection of OC. Similarly, we also observed the accumulation of these mainly pathogenic taxa in OC patients with adverse treatment outcomes compared to those without events and could also serve as potential indicators for predicting patients’ responses to treatment. These findings provide important insights into the potential use of the microbiome as indicators in (1) early detection of and screening for OC and (2) predicting patients’ response to treatment. Given the limited number of patients enrolled in the study, these results would need to be further investigated and confirmed in a larger study.

## Introduction

Ovarian cancer (OC) is the second most common gynecological malignancy and the fifth leading cause of death due to cancer in women in the United States. These statistics reflect mainly the late-stage diagnosis and poor prognosis of OC^1–3^. Ovarian cancer consists of two major types: Type I (30%) and Type II (70%) tumors^4^. Type I tumors are mainly slow growing low-grade serous, mucinous, endometrioid or clear cell^4^. Type II tumors, on the other hand, are usually aggressive high-grade serous, malignant mixed mesodermal, undifferentiated, or clear cell carcinomas^4^. Only about 20% of all OC incidence is explained by host genetic factors, mainly through germline mutations in the *BRCA1* and *BRCA2* genes^5–7^. With high incidence rates especially in developed countries, efforts in elucidating the cause of the remaining 80% of cases are focused on reproductive and environmental factors including age^8,9^, age at menarche^10^, parity^9,10^, breast feeding^10^, hormone replacement therapy (HRT)^11,12^, and oral contraceptive use^10,13^. A reduced risk of ovarian cancer has been associated with increasing age at menarche, increasing parity, breast feeding as well as oral contraceptive use^9,10,13^. Nulliparity and HRT on the other hand have been associated with increased risk of ovarian cancer^9–11^. While these and other risk factors contribute to the incidence of OC, they do not address the question of tumorigenic mechanism.

Due to its largely asymptomatic nature during early stages, resulting in more than two-thirds of OC patients presenting with advanced-stage disease^14^, it is critical to expand the search for specific indicators to help in early detection of the disease. Previous studies have shown significant influence of the microbiome in the etiology and progression of various cancers including *Helicobacter pylori* and gastric cancer^15^, Human Papillomavirus (HPV) and cervical cancer^16^ and *Fusobacterium* and colorectal cancer^17^. We have also shown significant association between *Porphyromonas somerae* and endometrial cancer^18,19^. Using our in-vitro invasion assays under hypoxic conditions, we further showed intracellular invasion of endometrial adenocarcinoma cells by *P. somerae*^20^. Several studies have also detected significant levels of HPV-16 and 18 in OC patients^21,22^. These results and many others provide evidence that the microbiome is an important source of potential indicators for early detection, diagnosis, or prognosis of the cancer. In this study, we sought to investigate the microbiome associated with OC and its potential role in detection, progression as well as prognosis of the disease.

## Results

To determine the impact of OC on the microbiome, we recruited women undergoing hysterectomy for OC or a benign gynecologic condition requiring hysterectomy. We then proceeded to compare the microbiome of patients with and without OC. Following that analysis, we focused on patients with OC and assessed the prognostic potential of the microbiome. We examined this impact using various α- (Inverse Simpson, and Shannon indices and observed ASVs) and β- (weighted, unweighted, and generalized UniFrac distances and Bray–Curtis) measures as well as differential abundance analysis. We report the α- and β-diversity measures with the most significant results in the main text and the remaining in the supplemental material.

### Patient demographics

We collected microbiome samples from a total of 64 women undergoing hysterectomy for either OC (n = 30) or a benign gynecologic condition (n = 34) at the Mayo Clinic in Rochester, MN (Table 1). Women with various benign gynecologic conditions were used as controls to characterize the microbiome specific to OC. The age (*p* = 0.672), menopausal state (*p* = 0.251) and body mass index (BMI) (*p* = 0.353) distributions were similar between both cohorts as shown in Table 1. We also show the results of tumor response to treatment as well as patients’ status two years and four years post-diagnosis (Table 2).

**Table 1:** Patient demographics.

	Benign (N = 30)	Ovarian Cancer (N = 34)	*p*-value
**Age at Diagnosis (years)**			
Median (Q1, Q3)	58.00 (50.25, 67.00)	61.000 (55.25, 68.00)	0.672
Range	37.00–83.00	37.00–84.00	
**BMI (kg/m**^**2**^**)**			
Median (Q1, Q3)	27.07 (23.92, 31.95)	27.21 (23.19, 29.76)	0.353
Range	19.21–40.82	20.48–41.22	
**Gravida**	2.7 (1.9)	2.2 (1.6)	0.282
**Parity**	2.6 (1.8)	1.9 (1.3)	0.075
**Ethnicity**			
Caucasian	29 (96.7%)	31 (91.2%)	0.402
Asian	1 (3.3%)	1 (2.9%)	
Other	0 (0%)	2 (5.9)	
**Menopausal status**			
Pre/Peri	12 (40%)	8 (23.5%)	0.251
Post	18 (60%)	26 (76.5%)	
**Vaginal pH**			
Normal (≤ 4.5)	6 (20%)	1 (2.9%)	0.805
High (> 4.5)	18 (60%)	7 (20.6%)	
Unknown	6 (20%)	26 (76.5%)	
**Smoking status**			
Never smoker	20 (66.7%)	24 (70.5%)	0.846
Previous smoker	7 (23.3%)	6 (17.7%)	
Current smoker	3 (10%0	4 (11.8%)	
**History of hypertension**			
Yes	18 (60%)	8 (23.5%)	**0.009**
No	12 (40%)	25 (73.5%)	
Unknown	0 (0%)	1 (2.9%)	
**History of diabetes**			
Yes, Type II	4 (13.3%)	4 (11.8%)	1.000
No	26 (86.7%)	29 (85.3%)	
Unknown	0 (0%)	1 (2.9%)	
**Indication for hysterectomy**			
Suspicion for OC	0 (0%)	32 (94.2%)	NA
Benign uterine conditions			
Abnormal bleeding	13 (43.4%)	1 (2.9%)	
Post-menopausal bleeding without biopsy prior to surgery with known BRCA 1 mutation	1 (3.3%)	0	
No Abnormal Bleeding	14 (46.7%)	0	
Uterine mass, pelvic fluid collection	1 (3.3%)	0	
Other—increasing CA 125	1 (3.3%)	0	
Unknown	0 (0%)	1 (2.9%)	

Significant values are in [bold].

Patient clinical characteristics. Data are presented as median (IQR) for continuous covariates and count (percent) for categorical covariates. Statistical significance assessed by t test for continuous covariates and chi-squared test for categorical covariates.

BMI , body mass index.

**Table 2:** Patient treatment response.

	Ovarian Cancer (N = 34)	
**Category**		
Malignant	32 (94.1%)	
Borderline	1 (2.9%)	
Borderline Malignant Mixed	1 (2.9%)	
**Malignancy type**		
Epithelial	25 (73.5%)	
Primary peritoneal	2 (5.9%)	
Fallopian tube	7 (20.6%)	
**Histology**		
Low grade serous	1 (2.9%)	
High grade serous	28 (82.4%)	
Mucinous	2 (5.9%)	
Endometrioid	1 (2.9%)	
Clear cell	2 (5.9%)	
**Stage**		
1	5 (14.7%)	
2	2 (5.9%)	
3	18 (52.9%)	
4	9 (26.5%)	
**Grade**		
0	1 (2.9%)	
1	3 (8.8%)	
2	1 (2.9%)	
3	29 (85.3%)	
**Surgery occurrence**		
Primary debulking	25 (73.5%)	
Interval debulking	3 (8.8%)	
Completion staging	6 (17.6%)	
**Debulking status**		
Optimal; no macroscopic disease	27 (79.4%)	
Optimal; macroscopic disease < 1 cm	6 (17.6%)	
Missing	1 (2.9%)	
**Chemo sequence**		
Neoadjuvant	3 (9.4%)	
first-line	29 (90.6%)	
N-Miss	2	
**Platinum chemo?**		
Yes	32 (100.0%)	
N-Miss	2	
**Taxane chemo?**		
No	1 (3.1%)	
Yes	31 (96.9%)	
N-Miss	2	
**Tumor response**		
Refractory	3 (12.0%)	
Resistant	1 (4.0%)	
Sensitive	21 (84.0%)	
N-Miss	9	
**Follow up (Status) post-diagnosis**	**Two years**	**Four years**
Alive; no event	17 (51.5%)	4 (19.0%)
Alive; event	11 (33.3%)	6 (28.6%)
Dead	5 (15.2%)	11 (52.4%)
N-Miss	1	13

Data are presented as count (percent).

N-Miss, number of missing information.

### The microbiome associated with ovarian cancer and benign uterine gynecologic conditions

**Figure 1:**
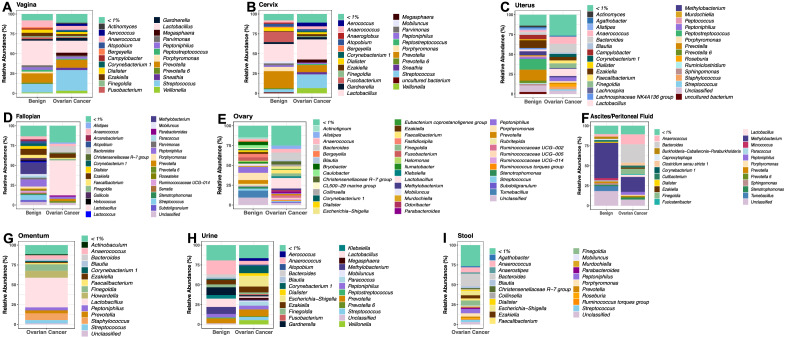
Genus-level microbial community composition (relative abundance) plots of patients with or without OC. (**A**) Vagina. (**B**) Cervix. (**C**) Uterus. (**D**) Fallopian tubes. (**E**) Ovary. (**F**) Ascites/Peritoneal fluid. (**G**) Omentum. (**H**) Urine. (**I**) Stool. Only microbial taxa present at a minimum of 1% relative frequency in at least one participant are shown for graphical clarity.

We sampled along the female reproductive tract (vagina, cervix, uterus, Fallopian tubes, ovaries), as well as ascites or peritoneal fluid, omentum (OC cohort only), urine, and stool (OC cohort only) to characterize the microbiomes of patients with either OC or a benign gynecologic condition. While the lack of omental and stool samples in the benign cohort did not allow for the comparisons between the two cohorts in these sample types, we were able to include the analysis of these samples in the OC cohort focusing on the impact of stage, grade, histology, and treatment response. The high throughput sequencing of the V3–V5 region of the 16S rRNA gene of all the 751 samples collected, including controls, yielded a total of 7076 ASVs. Our decontamination process (filtering out microbial taxa more abundant in the negative controls as well as present in more than one negative control) resulted in the removal of potential contaminants as shown in the abundance and relative abundance plots in Supplemental Fig. S1. The results of our taxonomic analysis showed that the microbiomes from the same body site of both benign and OC cohorts are generally dominated by the same microbial taxa to varying amounts (Fig. 1). For instance, the relative abundance of *Lactobacillus* in the vagina is only ~ 15% in the OC cohort compared to the ~ 30% in the benign cohort (Fig. 1). Several of these microbial taxa are also dominant across body sites. For example, *Lactobacillus* appears to be a dominant species in OC (vagina, cervix, uterus, Fallopian tubes, ovaries, and omentum) and benign (vagina, cervix, and urine) cohorts. *Ezakiella* also appear to be dominant across multiple sites (uterus, Fallopian tubes, urine, stool) in both OC and benign cohorts (Fig. 1). While *Peptoniphilus* is dominant in the cervix and ovaries of the benign cohort, *Porphyromonas* is particularly dominant in the Fallopian tubes and ovaries of the benign cohort and the stool of the OC cohort (Fig. 1). We also observed dominant levels of *Bacteroides* in the uterus, Fallopian tubes, ovary, ascites, and stool of the OC cohort (Fig. 1). Both *Prevotella* and *Streptococcus* are also dominant in the vagina and uterus of both OC and benign cohorts (Fig. 1).

### The distinguishing potential of the microbiome in ovarian cancer

#### Microbiome compositions of ovarian cancer patients differ significantly from those of patients with benign gynecologic conditions

**Figure 2:**
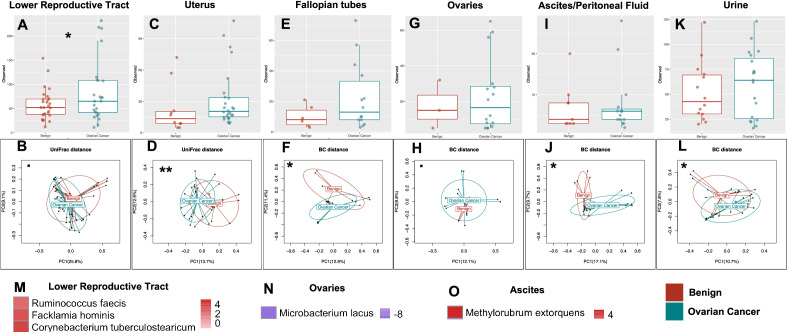
Bacterial community α- and β-diversities between patients with and without OC. Both α- (Observed ASVs) and β-diversity measures were compared. For α-diversity a Wald statistical test was performed and Observed ASVs was reported. For β-diversity, Bray–Curtis (BC) and unweighted UniFrac distance metrics were reported. The most significant metric is shown in each ordination plot. Lower reproductive tract (cervix and vagina), (**A**) Benign vs OC, α-diversity, *p* = 0.049, (**B**) Benign vs OC, β-diversity, unweighted UniFrac, *p* = 0.052. Uterus, (**C**) Benign vs OC, α-diversity, *p* = 0.907, (**D**) Benign vs OC, β-diversity, *p* = 0.004. Fallopian tube, (**E**) Benign vs OC, α-diversity, *p* = 0.201, (**F**) Benign vs OC, β-diversity, *p* = 0.025. Ovaries, (**G**) Benign vs OC, α-diversity, *p* = 0.766, (**H**) Benign vs OC, β-diversity, *p* = 0.088. Ascites/Peritoneal fluid, (**I**) Benign vs OC, α-diversity, *p* = 0.882, (**J**) Benign vs OC, β-diversity, *p* = 0.007. Urine, (**K**) Benign vs OC, α-diversity, *p* = 0.382. (**L**) Benign vs OC, β-diversity, *p* = 0.047. (**M–O**) Heatmaps showing the effect size (Log_2_ Fold Change) of the differentially abundant microbial taxa. White boxes reflect no fold change at FDR < 0.10. Analysis was adjusted for menopause status, and BMI. Samples rarefied prior to analysis. Analysis was adjusted for menopause status and BMI. *Groups are significantly different.

To further investigate the microbiome associated with OC, we summarized the differences in the microbiome composition between patients with or without OC using various α- (within-sample richness and evenness) and β- (between-sample) diversity measures. After adjusting for batch differences where necessary (See Methods), we compared the vaginal and cervical samples, and the results showed no significant differences (unweighted UniFrac: *p* = 0.814) between them in agreement with results from our previous studies^19,23^. We therefore combined the vaginal and cervical samples (lower reproductive tract, LRT) by adding sequence reads from both body sites for each patient in the rest of the present analysis. Our results revealed statistically significantly higher α-diversity in the LRT of the OC cohort compared to the benign cohort (Fig. 2A, Observed ASVs: *p* = 0.049; See Supplemental Fig. S2 for other metrics) which was not seen in the other body sites (uterus: Fig. 2C, Fallopian tubes: Fig. 2E, ovaries: Fig. 2G and urine: Fig. 2K). We also observed statistically significant β-diversity differences in the uterus (Fig. 2D, unweighted UniFrac: *p* = 0.004; Supplemental Fig. S3, weighted UniFrac: *p* = 0.028), Fallopian tube (Fig. 2F, Bray–Curtis: *p* = 0.025) and urine (Fig. 2L, Bray–Curtis, *p* = 0.047) between the benign and OC cohorts. Of note, we also observed differences in the β-diversity of the LRT (Fig. 2B, unweighted UniFrac: *p* = 0.052) and ovarian (Fig. 2H, Bray–Curtis, *p* = 0.088) microbiomes between the benign and OC cohorts that aligned with other organs but did not reach statistical significance. These differences resulted in general enrichment of several taxa, including *Corynebacterium tuberculostearicum*, *Facklamia hominis* and *Ruminococcus faecis* in the LRT and the depletion of *Microbacterium lacus* in the ovaries of the OC cohort (Fig. 2M and N; Supplemental Tables 1–3).

### Ovarian cancer microbiome according to stage, grade and histology

Following the general characterization of the microbiome from both OC and benign cohorts, we focused on characterizing the microbiome associated with the stage, grade, and histology of OC (Table 2).

#### Significant association between stage and the microbiome

**Figure 3:**
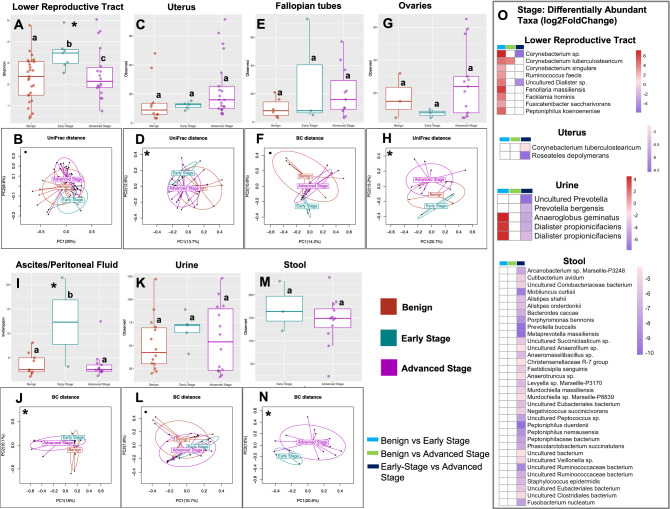
Bacterial community α- and β-diversities among patients with and without different stages of Ovarian Cancer (OC). Both α- and β-diversity measures were compared. For α-diversity a Wald statistical test was performed and Observed ASVs, Shannon Index and Inverse Simpson were reported. For β-diversity, Bray–Curtis (BC), unweighted, weighted, and generalized UniFrac distance metrics were reported. The most significant metric is shown in each ordination plot. Lower reproductive tract (cervix and vagina), (**A**) α-diversity: Benign vs Early stage (*p* = 0.019), Advanced stage (*p* = 0.025), (**B**) β-diversity: Benign vs Early stage (*p* = 0.061), Advanced stage (*p* = 0.059), Early vs Advanced stage (*p* = 0.065). Uterus, (**C**) α-diversity: Benign vs Early stage (*p* = 0.461), Advanced stage (*p* = 0.105), (**D**) β-diversity: Benign vs Early stage (*p* = 0.002), Advanced stage (*p* = 0.006), Early vs Advanced stage (*p* = 0.284). Fallopian tube, (**E**) α-diversity: Benign vs Early stage (*p* = 0.384), Advanced stage (*p* = 0.196), (**F**) β-diversity: Benign vs Early stage (*p* = 0.127), Advanced stage (*p* = 0.127), Early vs Advanced stage (*p* = 0.078). Ovaries, (**G**) α-diversity: Benign vs Early stage (*p* = 0.872), Advanced stage (*p* = 0.447), (**H**) β-diversity: Benign vs Early stage (*p* = 0.433), Advanced stage (*p* = 0.240), Early vs Advanced stage (*p* = 0.039). Ascites/Peritoneal fluid, (**I**) α-diversity: Benign vs Early stage (*p* = 0.054), Advanced stage (*p* = 0.010), (**J**) β-diversity: Benign vs Early stage (*p* = 0.166), Advanced stage (*p* = 0.028), Early vs Advanced stage (*p* = 0.091). Urine, (**K**) α-diversity: Benign vs Early stage (*p* = 0.310), Advanced stage (*p* = 0.380), (**L**) β-diversity: Benign vs Early stage (*p* = 0.175), Advanced stage (*p* = 0.086), Early vs Advanced stage (*p* = 0.566). Stool, (**M**) α-diversity: Benign vs Advanced stage (*p* = 0.302), (**N**) β-diversity: Early vs Advanced stage (*p* = 0.042). (**O**) Heatmaps showing the effect size (Log_2_ Fold Change) of the differentially abundant microbial taxa. White boxes reflect no fold change at FDR < 0.10. Analysis was adjusted for menopause status, and BMI. Samples rarefied prior to analysis. Wald statistical test with Q value cutoff = 0.1. *Groups are significantly different.

The presented results showed potentially important associations between OC stage and various measures of diversity. Specifically, a significant association between OC stage and α-diversity was observed across several sampling sites (Fig. 3 and Supplemental Fig. S4). Our results showed a statistically significant association of stage with the α-diversity of the LRT microbiome (Fig. 3A, Shannon, *p* = 0.034; Supplemental Fig. S4). The benign cohort had significantly lower (early-stage: Shannon, *p* = 0.019) and higher (advanced-stage: Shannon, *p* = 0.019) α-diversity than the OC cohort. We also showed significant association of stage with the β-diversity in multiple organs (Fig. 3; Supplemental Fig. S5). These include benign vs. early-stage (Fig. 3D, uterus: unweighted UniFrac, *p* = 0.002), benign vs. advanced-stage (Fig. 3D, uterus: unweighted UniFrac, *p* = 0.006), and early- vs. advanced-stage (Fig. 3H, ovaries: unweighted UniFrac, *p* = 0.039; Fig. 3N, stool: Bray–Curtis, *p* = 0.042). We also observed differences trending toward significant in the LRT (benign vs. early-stage: unweighted UniFrac, *p* = 0.061; benign vs. advanced-stage: unweighted UniFrac, *p* = 0.059; early- vs. advanced-stage: unweighted UniFrac, *p* = 0.065), Fallopian tube (early- vs. advanced-stage: Bray–Curtis, *p* = 0.078) and urine (benign vs. advanced-stage: Bray–Curtis, *p* = 0.086). Our differential abundance analysis results revealed general enrichment of several taxa in the LRT (*Peptoniphilus koenoeneniae*, *Facklamia hominis*, *Ruminococcus faecis*, *Fenollaria massiliensis*) and urine (*Dialister propionicifaciens* and *Anaeroglobus geminatus*) of patients in both early- and advanced-stages of OC compared to the benign cohort (Fig. 3O; Supplemental Tables 1–2, and 4–5). We however observed general depletion of microbial taxa in the LRT (*Corynebacterium *sp. and *Dialister* sp.), uterus (*Corynebacterium tuberculostearicum* and *Roseateles depolymerans*), urine (*Prevotella bergensis*, *Dialister propionicifaciens* and *Anaeroglobus geminatus*) and stool (*Peptoniphilus duerdenii*, *Prevotella buccalis*, *Mobiluncus curtisii*, *Porphyromonas bennonis* and *Alistipes shahii*) of advanced-stage OC patients in comparison to early-stage OC patients (Fig. 3O; Supplemental Tables 1–2 and 6).

#### Significant association between grade and the microbiome

**Figure 4:**
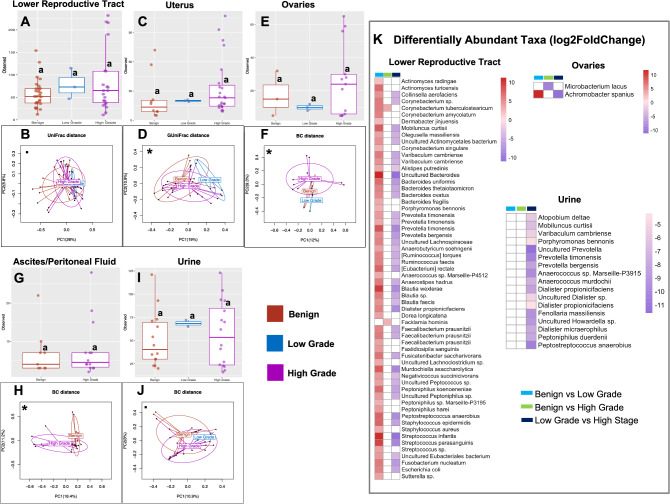
Bacterial community α- and β-diversity among patients with and without different grades of OC (OC). Both α- and β-diversities measures were compared. For α-diversity a Wald statistical test was performed and Observed ASVs was reported. For β-diversity, Bray–Curtis (BC), unweighted, and generalized UniFrac distance metrics were reported. The most significant metric is shown in each ordination plot. Lower reproductive tract (cervix and vagina), (**A**) α-diversity: Benign vs Low grade (*p* = 0.221), High grade (*p* = 0.997), (**B**) β-diversity: Benign vs Low grade (*p* = 0.195), High grade (*p* = 0.087), Low grade vs High grade (*p* = 0.406). Uterus, (**C**) α-diversity: Benign vs Low grade (*p* = 0.400), High grade (*p* = 0.159), (**D**) β-diversity: Benign vs Low grade (*p* = 0.023), High grade (*p* = 0.014), Low grade vs High grade (*p* = 0.019). Ovaries, (**E**) α-diversity: Benign vs Low grade (*p* = 0.972), High grade (*p* = 0.552), (**F**) β-diversity: Benign vs Low grade (*p* = 0.350), High grade (*p* = 0.067), Low grade vs High grade (*p* = 0.045). Ascites/Peritoneal fluid, (**G**) α-diversity: Benign vs High grade (*p* = 0.536), **(H)** β-diversity: Benign vs High grade (*p* = 0.016). Urine, (**I**) α-diversity: Benign vs Low grade (*p* = 0.490), High grade (*p* = 0.534), (**J**) β-diversity: Benign vs Low grade (*p* = 0.615), High grade (*p* = 0.056), Low grade vs High grade (*p* = 0.717). (**K**) Heatmaps showing the effect size (Log_2_ Fold Change) of the differentially abundant microbial taxa. White boxes reflect no fold change at FDR < 0.10. Analysis was adjusted for menopause status, and BMI. Samples rarefied prior to analysis. Wald statistical test with Q value cutoff = 0.1. *Groups are significantly different.

While we did not observe any significant association of grade with α-diversity, our results revealed significant association of grade with β-diversity in the uterine and ovarian microbiomes (Fig. 4; Supplemental Fig. S6 and S7). These include significant differences between benign vs. low-grade (Fig. 4D, uterus: generalized UniFrac, *p* = 0.023), benign vs. high-grade (Fig. 4D, uterus: generalized UniFrac, *p* = 0.014), and low- vs. high-grade (Fig. 4D, uterus: generalized UniFrac, *p* = 0.019; Fig. 4F, ovaries: Bray–Curtis, *p* = 0.045) OC patients. Differences which were not quite significant were also observed between benign and high-grade OC patients in the LRT (Fig. 4B, unweighted UniFrac, *p* = 0.087), ovaries (Fig. 4F, Bray–Curtis, *p* = 0.067) and urine (Fig. 4J, Bray–Curtis, *p* = 0.056). The results of the differential abundance analysis revealed general enrichment of several taxa in the LRT of both low- and high-grade patients compared to the benign cohort (Fig. 4K; Supplemental Tables 1–2, and 7–8). The enriched taxa include *Streptococcus infantis*, *Fusobacterium nucleatum*, *Varibaculum cambriense*, *Escherichia coli*, *Faecalibacterium prausnitzii*, and *Bacteroides fragilis*. Comparing LRT microbiome of low-grade OC patients to that of high-grade OC patients, however, results in the depletion of these microbial taxa in the high-grade OC patients (Fig. 4K; Supplemental Tables 1–2, and 9). We also observed similar trends in the urinary microbiome with general depletion of microbial taxa in high-grade OC patients compared to the low-grade OC patients. A few examples of the depleted taxa include *Peptostreptococcus anaerobius*, *Mobiluncus curtisii*, *Dialister propionicifaciens*, *Peptoniphilus. bennonis*, and *Atopobium deltae* (Fig. 4K; Supplemental Tables 1–2, and 9).

#### Significant association between histology and the microbiome

**Figure 5:**
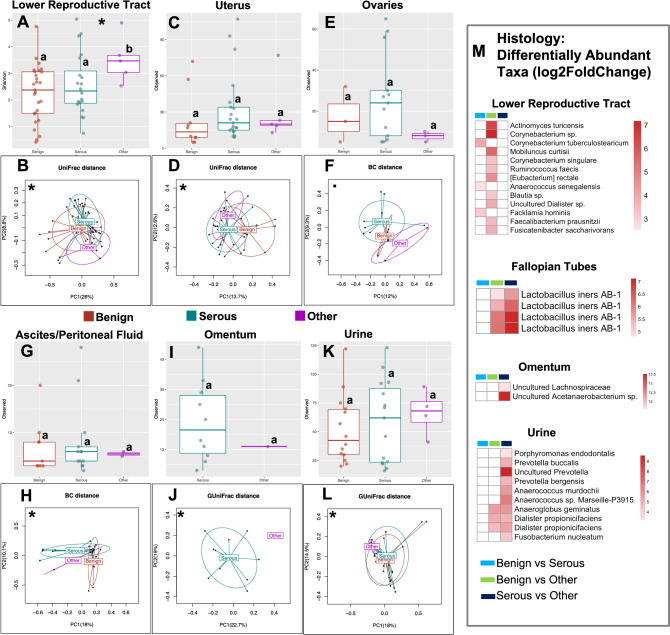
Bacterial community α- and β-diversities among patients with and without different histology of OC (OC). Both α- and β-diversities measures were compared. For α-diversity a Wald statistical test was performed and Observed ASVs was reported. For β-diversity, Bray–Curtis (BC), unweighted, and generalized UniFrac distance metrics were reported. The most significant metric is shown in each ordination plot. Lower reproductive tract (cervix and vagina), (**A**) α-diversity: Benign vs serous (*p* = 0.021), others (*p* = 0.859), (**B**) β-diversity: Benign vs serous (*p* = 0.038), others (*p* = 0.238), serous vs others (*p* = 0.275). Uterus, (**C**) α-diversity: Benign vs serous (*p* = 0.767), others (*p* = 0.459), (**D**) β-diversity: Benign vs serous (*p* = 0.002), others (*p* = 0.123), serous vs others (*p* = 0.400). Ovaries, (**E**) α-diversity: Benign vs serous (*p* = 0.918), others (*p* = 0.234), (**F**) β-diversity: Benign vs serous (*p* = 0.051), others (*p* = 0.433), serous vs others (*p* = 0.138). Ascites/Peritoneal fluid, (**G**) α-diversity: Benign vs serous (*p* = 0.636), others (*p* = 0.807), (**H**) β-diversity: Benign vs serous (*p* = 0.019), others (*p* = 0.667), serous vs others (*p* = 0.571). Omentum, (**I**) α-diversity: Serous vs others (*p* = 0.377), (**J**) β-diversity: serous vs others (*p* = 0.003). Urine, (**K**) α-diversity: Benign vs serous (*p* = 0.360), others (*p* = 0.911), (**L**) β-diversity: Benign vs serous (*p* = 0.329), others (*p* = 0.048), serous vs others (*p* = 0.129). (**M**) Heatmaps showing the effect size (Log_2_ Fold Change) of the differentially abundant microbial taxa. White boxes reflect no fold change at FDR < 0.10. Analysis was adjusted for menopause status, and BMI. Samples rarefied prior to analysis. Wald statistical test with Q value cutoff = 0.1. *Groups are significantly different.

Consistent with results obtained from stage, histological features of OC are significantly associated with both α-diversity and β-diversity in multiple body sites (Fig. 5; Supplemental Figs. S8 and S9). There was a significant overall association of histology with the α-diversity of the LRT microbiome (Fig. 5A, Shannon, *p* = 0.045), with a significantly lower α-diversity in the benign cohort compared to other OC histologies (Shannon, *p* = 0.015). The β-diversity analysis results showed significant differences between the patients with benign lesions and serous OC (Fig. 5B, LRT: unweighted UniFrac, *p* = 0.038; Fig. 5D, uterus: unweighted UniFrac; *p* = 0.002), between patients with benign conditions and other histologies (Fig. 5L, urine: generalized UniFrac, *p* = 0.048) as well as between serous and other histologies (Fig. 5J, omentum: generalized UniFrac). We also observed differences between the microbiota of ovaries from patients with benign lesions vs serous OC that aligned with other organs but did not reach statistical significance (Bray–Curtis, *p* = 0.051). Results from the differential abundance analysis revealed general enrichment of several microbial taxa in the LRT, Fallopian tube, omentum, and urine of OC patients with serous and other histologies compared to the benign cohort (Fig. 5M; Supplemental Tables 1–2, and 10–11). These enriched microbial taxa include *Facklamia hominis*, *Anaerococcus senegalensis*, *Lactobacillus iners*, and *Actinomyces turicensis*. Within the OC patient cohort, the results of the differential abundant analysis also showed enrichment of microbial taxa including *Lactobacillus iners*, *Fusobacterium nucleatum*, *Prevotella buccalis*, and *Dialister propionicifaciens*, in patients with other OC histologies in comparison to the serous OC patients (Fig. 5M; Supplemental Tables 1–2, and 12).

### Microbiome prognostic potential for ovarian cancer treatment

#### The ovarian cancer microbiome is prognostic of treatment response

**Figure 6:**
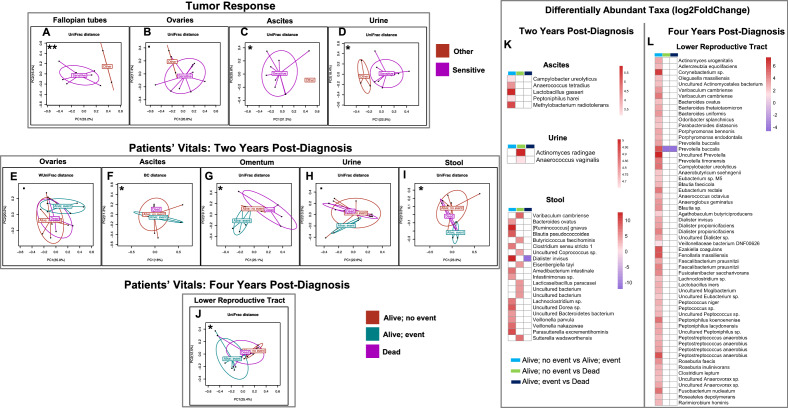
β-diversities measures were compared. For β-diversity, Bray–Curtis (BC), unweighted, and weighted, UniFrac distance metrics were reported. The most significant metric is shown in each ordination plot. (**A–D**) Bacterial community β-diversity between OC patients with sensitive vs other (resistant/refractory) tumor response. Fallopian tube, (**A**) β-diversity: sensitive vs other (*p* = 0.003). Ovaries, (**B**) β-diversity: sensitive vs other (*p* = 0.073). Ascites, (**C**) β-diversity: sensitive vs other (*p* = 0.021). Urine, (**D**) β-diversity: sensitive vs other (*p* = 0.015). (**E–I**) Bacterial community β-diversity among OC patients with different status two years post-diagnosis. Ovaries, (**E**) β-diversity: Alive, no event vs alive, event (*p* = 0.073), Alive, no event vs dead (*p* = 0.761), Alive, event vs dead. (*p* = 0.437). Ascites, (**F**) β-diversity: Alive, no event vs alive, event (*p* = 0.573), Alive, no event vs dead (*p* = 0.029), Alive, event vs dead (*p* = 0.250). Omentum, (**G**) β-diversity: Alive, no event vs alive, event (*p* = 0.273), Alive, no event vs dead (*p* = 0.350), Alive, event vs dead (*p* = 0.010). Urine, (**H**) β-diversity: Alive, no event vs alive, event (*p* = 0.088), Alive, no event vs dead (*p* = 0.347), Alive, event vs dead (*p* = 0.356). Stool, (**I**) β-diversity: Alive, no event vs alive, event (*p* = 0.063), Alive, no event vs dead (*p* = 0.601), Alive, event vs dead (*p* = 0.050). (**J**) Bacterial community β-diversity among OC patients with different status four years post-diagnosis. Lower reproductive tract (cervix and vagina), (**J**) β-diversity: Alive, no event vs alive, event (*p* = 0.017), Alive, no event vs dead (*p* = 0.568), Alive, event vs dead (*p* = 0.058). (**K**–**L**) Heatmaps showing the effect size (Log_2_ Fold Change) of the differentially abundant microbial taxa. White boxes reflect no fold change at FDR < 0.10. Analysis was adjusted for menopause status, and BMI. Samples rarefied prior to analysis. Wald statistical test with Q value cutoff = 0.1. *Groups are significantly different.

Because the microbiome samples were collected from treatment naïve patients, we also investigated the role of microbiome in treatment response to better understand the prognostic potential of the microbiome at the time of hysterectomy. We explored outcome data including tumor response, patients’ status two years and four years post-diagnosis (Table 2). Our results showed significant association of the tumor response with both α-diversity and β-diversity in multiple body sites (Fig. 6A–D; Supplemental Figs. S10–S11). We found a significantly lower α-diversity (Inverse Simpson, *p* = 0.044) in the omental microbiome of patients who had chemotherapy sensitive OCs in comparison to those who did not (Supplemental Fig. S10). Our β-diversity results also showed significant differences between patients with chemotherapy sensitive OCs (Fallopian tube: unweighted UniFrac, *p* = 0.003; urine: unweighted UniFrac, *p* = 0.015) compared to refractory/resistant (other) OCs (Fig. 6A and D; Supplemental Fig. S11). These differences however did not result in differentially abundant microbial taxa between the two groups (Fig. 6; Supplemental Tables 1–2, and 13). We further analyzed the potential of the microbiome to predict patients’ status two years and four years post-diagnosis (Table 2). Our results showed significant differences in both α-diversity and β-diversity in multiple body sites (Fig. 6E–J; Supplemental Figs. S12–S15). We report a significantly higher α-diversity (uterus: Shannon, *p* = 0.038) in patients who were alive with no adverse events after two years compared to those who were deceased (Supplemental Fig. S12). These differences are also seen in the β-diversity results (Fig. 6; Supplemental Fig. S13) with significant differences between patients who were alive but experienced adverse events and those who were deceased two years post-diagnosis (Fig. 6G, omentum: unweighted UniFrac, *p* = 0.010; Fig. 6I, stool: unweighted UniFrac, *p* = 0.050). We also observed significant differences in the β-diversity of patients who were alive with no adverse events and those who experienced adverse events (Fig. 6J, LRT: unweighted UniFrac, *p* = 0.017; Supplemental Figs. S14–S15) 4 years post-diagnosis. While not statistically significant, we also observed differences between patients who were alive with adverse events and those who were deceased (Fig. 6J, LRT: unweighted UniFrac, *p* = 0.058). Our differential abundance analysis identified several differentially enriched microbial taxa in the urine and stool of patients who were alive but experienced adverse events and those who were deceased compared to those who were alive with no adverse events two years post-diagnosis (Fig. 6K Supplemental Tables 1–2, and 14–16). Examples include *Lactobacillus gasseri*, *Diasliter invisus.*, *Blautia pseudococcoides*, *Veillonella nakazawae*, *Bacteroides ovatus*, *Butyricicoccus faecihominis* and *Sutterella wadsworthensis*, (Fig. 6K; Supplemental Tables 1–2, and 14–16). The LRT microbiomes of patients who were alive with adverse events had generally enriched taxa (*Lactobacillus iners*, *Fenollaria massiliensis*, *Ezakiella coagulans*, and *Campylobacter ureolyticus*, and *Actinomyces urogenitalis*) in comparison to those who were alive without event four years post-diagnosis (Fig. 6L; Supplemental Tables 1–2, and 17). We further observed general depletion of *Prevotella bucalis* in patients who were deceased compared to those who were alive with or without adverse events (Fig. 6L; Supplemental Tables 1–2, and 18–19).

#### Microbiome composition of malignant versus benign peritoneal fluid

The volume of ascites at initial surgery of epithelial OC has been shown to be an important clinical parameter in the prognosis of the disease^24^. We therefore compared the peritoneal fluid microbiome of patients with or without OC to characterize the microbiome composition associated with ascites. Here we compared the properties of peritoneal fluid from patients with OC vs. those without OC. Our taxonomic analysis results showed that in addition to both the benign cohort and OC cohort having peritoneal fluid microbiomes dominated by *Methylobacterium*, *Anaerococcus*, and *Stenotrophomonas*, the OC cohort was also dominated by *Bacteroides*, *Finegoldia*, *Lactobacillus* and *Peptoniphilus*; and the benign cohort by *Tumebacillus*, *Micrococcus* and *Prevotella* (Fig. 1F). While this did not result in significant differences in the α-diversity (Fig. 2I) between these two cohorts, our results showed significant differences in β-diversity in the peritoneal fluid between patients with OC and those without (Fig. 2J, Bray–Curtis, *p* = 0.007). These reflected the enrichment of *Methylorubrum extorquens* in the OC cohort (Fig. 2, Supplemental Tables 1–3). Following these analyses, we also characterized the peritoneal fluid microbiomes associated with the stage, grade, and histology of OC compared to the benign conditions at the time of hysterectomy (Table 2). We observed significant differences in both α-diversity and β-diversity in malignant ascites versus peritoneal fluid from patients with benign conditions. These include significant differences in α-diversity between samples from patients with benign conditions vs. advanced-stage OC patients (Fig. 3I; Bray–Curtis, *p* = 0.014) with enriched *M. extorquens* in the OC patients (Supplemental Tables 1–2, and 5). Our results also showed significant differences in β-diversity between the patients with benign conditions and high-grade OC patients (Fig. 4H; Bray–Curtis, *p* = 0.016). We also observed significant differences in the in β-diversity between the patients with benign conditions vs. serous OC patients (Fig. 4; Bray–Curtis, *p* = 0.019), with enriched *M. extorquens* in the OC patients (Supplemental Tables 1–2, and 10). Finally, we also explored the prognostic potential of the peritoneal fluid in treatment response (Fig. 6F; Supplemental Tables 1–2, and 13–14). Our results showed significant differences in the β-diversity of patients with sensitive tumor response compared to others (Fig. 6F; unweighted UniFrac, *p* = 0.022), with enriched *Anaerococcus tetradius* in patients who did not experience sensitive tumor response (Fig. 6M, Supplemental Tables 1–2 and 13). We also showed significant differences in the β-diversity of the patients who were alive without adverse events and those who were deceased two years post-diagnosis (Fig. 6F; Bray–Curtis, *p* = 0.029). A few microbial taxa including *A. tetradius*, *Peptoniphilus harei*, *Methylobacterium radiotolerans*, and *Lactobacullus gasseri* were also found enriched in patients who were alive with adverse events compared those who were alive with no adverse events two years post-diagnosis (Fig. 6M, Supplemental Tables 1–2, and 14).

## Discussion

OC, which is the second most common gynecological malignancy and the fifth leading cause of death due to cancer in women in the United States, is most often diagnosed at advanced stage, contributing to its very poor prognosis. It is, therefore, critical to identify potential indicators to aid in early detection as well as prediction of treatment response. In this study, we characterized the RT, ascites/peritoneal fluid, omental, urinary and stool microbiome compositions of patients diagnosed with a variety of benign uterine conditions warranting a hysterectomy (abnormal bleeding, uterine mass, and pelvic fluid collection) or an OC diagnosis (serous, clear cell, mucinous and mixed histologies).

**Figure 7:**
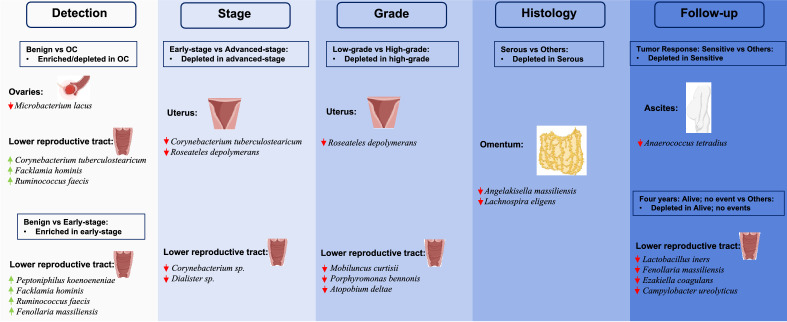
A summary of differentially abundant microbial species observed in the various comparisons carried out in the study. Red down arrows () indicate depletion while green up arrows () indicate enrichment. Created with BioRender.com.

Changes in relative abundance of specific groups of microbial taxa have been reported to damage DNA, resulting in genetic dysregulation and initiation of tumorigenesis^25^. Our results showed significant differences in both α- and β-diversities between the benign and OC cohorts due to changes in relative abundance of specific microbes between the two cohorts. For instance, our observation of relatively lower abundance of *Lactobacillus* species in the LRT of the OC cohort compared to the benign cohort is consistent with the lower abundance of *Lactobacillus* species reported in the cervicovaginal microbiome of OC patients compared to healthy and benign controls^26^, particularly in women younger than 50 years of age^27^. Other examples include relatively higher abundance of *Streptococcus*, *Aeroccocus*, *Veillonella* and *Megasphaera* and lower abundance of *Fusobacterium* in LRT of the OC cohort. *Streptococcus* and *Veillonella* were both reported to have been enriched in the lower airways of lung cancer patients, resulting in the up-regulation of extracellular signal-regulated kinase (ERK) and phosphoinositide 3-kinase (PI3K) signaling pathways^28^. Our results also revealed a higher relative abundance of *Bacteroides* in the uterus, Fallopian tubes, ovaries, and ascites of the OC cohort. Enterotoxigenic *B. fragilis* has been shown to induce colon tumors in multiple intestinal neoplasms in mice^29^. These results are consistent with previous reports of microbial associations with various types of cancers^25–27,30–32^. These differences in the relative abundance as well as significantly higher α-diversity resulted in statistically significant enrichment in several pathogenic bacteria in the LRT of the OC cohort (Fig. 7). For instance, *C. tuberculostearicum*, which has been isolated from patients with mastitis^33^ and clinical samples from patients who exhibited multi-drug resistance^34^, was enriched in the LRT of the OC cohort. Another known pathogen, *F. hominis* shown to cause bacteremia was also significantly enriched in the LRT of the OC cohort^35,36^. While most of the microbial taxa enriched in the LRT of the OC are known pathogens, we also observed enrichment of *R. faecis*, that have been shown to alleviate liver damage nonalcoholic fatty liver disease (NAFLD) mice^37^. These results reveal an overwhelming presence of known pathogens in the LRT of the OC cohort that could play important roles in early detection of OC.

To probe the diagnostic potential of the microbiome in OC, we focused on the differences in the microbiome within the OC cohort (stage, grade, and histology) as well as compared to the benign cohort. The results show significant differences between the benign cohort and early- and advanced-stage disease. These differences resulted in the enrichment of several known pathogens in the LRT and urinary microbiomes of patients in various stages of OC in comparison to the benign cohort (Fig. 7). Several of the enriched taxa, including *C. tuberculostearicum*, *C. singular*, *P. koenoeneniae* and *F. hominis*, are shown to cause bacterial vaginosis, urinary tract infections and bacteremia^33,35,36,38^. The observed general depletion of several known pathogens in the LRT, uterus, urine, and stool of advanced-stage patients compared to early-stage underscores the vital role these differentially enriched microbial taxa could play in the early detection and/or diagnosis of OC in early-stage OC patients (Fig. 7). They appear to accumulate during the early stages of the disease and become depleted as the cancer advances. These results provide evidence for the importance of investing in longitudinal sampling to further understand when this change occurs and if we can detect the difference earlier for clinical purposes. With more than two-thirds of OC patients diagnosed at advanced-stage, early diagnosis could result in 5-year relative survival rate of about 93%^39^. Like stage, there was general enrichment in several known pathogens in the LRT of patients with various grades and histology of OC in comparison to the benign cohort. One of such taxa is a known pathogen, *M. curtisii*, which has been shown to be associated with recurrence of bacterial vaginosis due to resistance to metronidazole, was also significantly enriched in the LRT of the low-grade OC cohort^40^. We also show significant enrichment of *Eubacterium rectale*, which has been reported to function as a “driver” bacterium in the initiation of colorectal cancer^41^, in the LRT of the low-grade OC cohort. Some of the other enriched taxa have also been associated with cancers including oral, bladder and colorectal (*F. nucleatum*), endometrial (*Porphyromonas* and *Peptoniphilus*) and breast (*Aerococcus*) cancers^17,18,30,42,43^. While most of the microbial taxa enriched in the LRT of the OC were known pathogens, we also observed enrichment in others such as *Faecalibacterium prausnitzii*, *Dorea longicatena* and *Blautia spp.* that have been shown to have probiotic properties^44–46^. Consistent with the results from stage, the general depletion of these known pathogens in the high-grade OC patients compared to the low-grade OC patients further emphasizes their importance in early detection as well as diagnosis of OC. In general, these results show the accumulation of mostly detrimental microbes especially in early-stage, low-grade OC patients which appear to decrease in advance-stage, high-grade OC patients. These results need to be further investigated in a larger longitudinal study to better understand the composition of these detrimental microbes, timing of their accumulation and when the decrease begins for earlier and better detection and diagnosis of OC.

Several studies have reported results suggesting the role of microbes in the efficacy of cancer therapies^47–49^. For instance, the chemotherapeutic drug gemcitabine has been shown to be metabolized by bacteria, including *Mycoplasma hyorhinis*, into its inactive form in murine colon cancer models^47^. Yamamura et al.^48^ also reported an association between high burdens of *F. nucleatum* in clinical esophageal squamous cell carcinomas and poor recurrence-free survival. Similarly, *M. curtisii* has been shown to be associated with recurrence of bacterial vaginosis due to resistance to metronidazole^40^. We leveraged follow-up data from the OC cohort on response to treatment to examine the relationship between the OC-associated microbiome and response to treatment at two years and four years post-diagnosis. Our findings revealed the enrichment of several known pathogens, including *Bacteroides ovatus*, *V. parvula*, and *A. christensenii*^50–52^, in the LRT and stool of patients with adverse outcomes. For instance, spinal infection with *V. parvula*, which has been shown to be resistant to tetracycline, vancomycin, aminoglycosides, and ciprofloxacin, was reported in a man with sinus malignancy^50^. We also showed the enrichment of several *Dialister* species including *D. invisus*, *D. micraerophilus* and *D. propionicifaciens* in patients with adverse outcomes. Morio et al.^53^ reported decreased susceptibilities of several *Dialister* isolates from clinical samples to piperacillin, metronidazole, macrolides, fluoroquinolones, and rifampin suggestive of possible multi-drug resistance in these patients. The results emphasize the potential role of these microbes in patients’ response to treatment and as well as predicting how patients will respond to OC treatment.

Putting all the results together, there is a clear pattern of general enrichment of known pathogenic microbial taxa in the OC patients in comparison to the patients undergoing hysterectomy for benign indications. This general enrichment of pathogenic taxa is further seen in early- and advanced-stage, low- and high-grade as well as serous OC and other OC histologies compared to patients with benign conditions. However, we also see general depletion of these pathogenic microbial taxa in patients with advanced-stage and high-grade OC compared to patients with early-stage and low-grade OC. These results suggest that the accumulation of the pathogenic taxa is highest in low grade, early stage of the disease which presents an opportunity for early detection. A focus on the treatment outcomes for OC patients also shows the enrichment of pathogenic microbial taxa in the patients with adverse outcomes compared those who alive with no events. Like stage and grade, these taxa are depleted in samples from patients who succumbed to OC compared those who are alive but experienced adverse events. These results further suggest that the accumulation of these pathogenic taxa could potentially lead to adverse treatment outcomes and present an opportunity for better treatment options that account for these pathogenic taxa.

We acknowledge the limitations of the number of patients enrolled in this study present. These results therefore need to be further explored and confirmed in a larger study.

## Conclusions

Our study revealed a distinct microbiome signature in patients with OC compared to patients with benign gynecological conditions. We identified several differentially abundant microbial taxa between the benign cohort versus early- and advanced-stage OC patients that could play vital roles in early detection of and screening for OC. Finally, with differentially abundant microbial taxa, we also showed that the microbiome of patients before treatment could potentially predict their response to treatment. These results need to be further investigated and confirmed in a larger study.

## Methods

### Ethics statement

Protocols #12-004445 (approved 8/13/2012) and #15-007679 (approved 1/22/2016) for patient enrollment with written informed consent were approved by the Mayo Clinic Institutional Review Board (IRB). Patients were recruited from 01/05/2013 to 5/7/2018 using methods and procedures that were in accordance with the Mayo Clinic IRB guidelines and regulations.

### Patient enrollment

A total of 64 women who were 18 years of age or older and undergoing hysterectomy for OC (N = 34) or a benign gynecologic condition (N = 30) requiring hysterectomy by standard surgical procedures at the Division of Gynecologic Surgery at Mayo Clinic in Rochester, MN, were included in this study (Table 1). The indication for hysterectomy was an inclusion criterion and most of the patients also received salpingo-oophorectomy. We excluded women if they were pregnant or nursing, had antibiotic treatment in the two weeks preceding surgery, or if morcellation was used during the hysterectomy for any reason (e.g., size of the uterus). Patients provided stool samples the day preceding or the day of the surgery while urine specimens were collected through a catheter in the operating room (OR). The vaginal and cervical samples were collected by the surgeon in the OR immediately preceding the betadine vaginal scrub. Ascites/peritoneal fluid was collected shortly after incision by the surgical team. The remaining samples (uterine, Fallopian tubes, ovarian and omental) were collected in the Pathology Laboratory within minutes of surgical extraction, by a Pathologist Assistant using aseptic technique.

### Treatment response data collection

Given that samples for this study were collected from treatment naïve patients, we also investigated the role of the microbiome on treatment response. We obtained various treatment outcome data on all patients including tumor response to treatment and patient status, at approximately two years and four years post-diagnosis (Table 2). For samples with primary debulking/completion staging followed by platinum/taxane treatment, the following definitions for tumor response were used: (1) Refractory: recurrence while receiving the chemotherapy or within four weeks of the last dose of therapy or Persistent Disease = “yes”, (2) Resistant: recurrence from 4 weeks to 6 months after the last dose of chemotherapy, and (3) Sensitive: no recurrence or recurrence more than 6 months after last dose of chemotherapy. Regarding status post-diagnosis, patients were either alive (with or without any adverse event such as cancer recurrence) or deceased after suffering adverse event(s).

### Sample collection

#### Operating room collection

Vaginal, cervical, urine, and ascites/peritoneal lavage samples were collected as described previously^18^. Briefly, vaginal, and cervical swabs were collected using two sterile Dacron swabs by the surgeon (with guidance on site by the research team) immediately after anesthesia administration but before the standard pre-operative betadine scrub and placed in sterile Tris–EDTA and transported on dry ice to storage at − 80 °C. Urine was obtained during the surgery through a catheter. Ascites was obtained during surgical aspiration following incision immediately following ascites sample collection for diagnostic cytology procedures. If no ascites was present, sterile saline was flushed into the abdominal cavity as standard surgical procedure. That clinical waste aspirate was collected for research use and microbiome analysis.

#### Pathology laboratory collection

Uterine, Fallopian tube, ovarian, and omentum samples were collected as described previously^18^. Briefly, the uterus, Fallopian tube, ovaries, and omentum were transported (under 2 min) in a sterile bag at room temperature to the pathology lab for processing under sterile conditions. The organs were processed at the grossing station by the research team after sterilization. Following the bilateral cut and splaying of the uterus (by the pathologist’s assistant (PA)), whole uterine swabs (Dacron) and scrapes (sterilized pap smear spatulas) were collected. Samples necessary for diagnosis were then collected by the PA and research dedicated biopsies were collected immediately after diagnostic procedures were complete.

### Stool samples

Patients were requested to provide a stool sample collected within a 24 h period of their scheduled surgery. When received, samples were stored at − 80 °C until processing.

### Sample processing and genomic DNA extraction

Samples were processed and genomic DNA sequenced as described previously^18^. Briefly, we thawed and vortexed the swab and scrape samples to mix any settled material and then centrifuged to pellet bacterial cells while a sterile pestle was used to macerate the biopsy samples. This was followed by genomic DNA extraction from approximately 100 mg of tissue using the MoBio PowerSoil DNA Isolation Kit (MoBio Laboratories, Inc., Carlsbad, CA, USA) according to the manufacturer’s protocol. The MP FastPrep (MP Biomedicals, Solon, OH, USA) was used for 60 s at 6.0 m/s to obtain a more effective and rapid lysis of the cells. We measured the DNA concentration using High Sensitivity Qubit (Life Technologies Corporation, Carlsbad, CA, USA). In addition to the samples, controls (Blank, PCR negative control, PCR positive control *Geobacillus* and TE *Geobacillus*) of the DNA extraction were performed and are shown in Supplemental Fig. 1.

### Sequencing

Samples were sequenced as described previously^19^. Briefly, we amplified the V3-V5 region of the 16S rRNA gene via a two-step polymerase chain reaction (PCR) protocol using the following universal forward (V3_357F: 5′GTCCTACGGGAGGCAGCAG3′) and reverse (V5_926R: 5′CCGTCAATTCMTTTRAGT3′) primers^54^ followed by the addition of Illumina flow cell adaptors containing indices^55^. Following the primary PCR, the products were diluted (1:100) in PCR grade water for secondary PCR reactions using V3_357F and V5_926R primers modified with Nextera adaptors developed in collaboration with the University of Minnesota Genomic Center in Minneapolis, MN.

V3_341F_Nextera: TCGTCGGCAGCGTCAGATGTGTATAAGAGACAGCCTACGGGAGGCAGCAG.

V5_926R_Nextera: GTCTCGTGGGCTCGGAGATGTGTATAAGAGACAGCCGTCAATTCMTTTRAGT.

A detailed procedure for both primary and secondary PCR is given in Walsh et al.^19^. This was followed by dilution, normalization, and pooling of the PCR products, which were then concentrated and cleaned up using 1.8X AMPureAP beads (Beckman Coulter, Brea, CA). After quantification using a Quant-It dsDNA HS assay kit (Thermo Fisher Scientific Inc., Waltham, MA), the sequence pool was assessed for purity and the presence of 725 bp peak (± 20%) using a 2200 TapeStation system and D1000 Screen tape/reagents (Agilent Technologies, Santa Clara, CA). The pooled 16S amplicons were sequenced using the MiSeq 600 cycle v3 kit (Illumina, San Diego, CA) and MCS v2.6.1 after quantification using KAPA SYBR FAST qPCR kit (KAPA Biosystems, Woburn, MA), dilution and denaturing.

### Sequence analysis

The sequenced reads were processed using the quantitative insights into microbial ecology (QIIME2) as follows: Using sample-specific barcodes assigned during sequencing, we demultiplexed all reads in QIIME2-2020.11^56^. This was followed by quality control, denoising, chimera removal and amplicon sequence variants (ASVs) generation for each sequence run using the Divisive Amplicon Denoising Algorithm (DADA2)^57^ within QIIME2. Taxonomy was then assigned at 99% similarity based on the SILVA taxonomy and reference database (SILVA_132_QIIME_release)^58^ and a rooted phylogenetic tree built using the “align-to-tree-mafft-fasttree” pipeline from QIIME2. To rigorously exclude potential contaminants, we imported the QIIME2 output files into R (R software, version 4.0.3, https://www.r-project.org) and filtered out taxa more abundant in the negative controls than samples using the R package decontam version 1.10.0. We followed this with further removal of any taxon that appeared in more than one negative control.

### Sequencing outcome

We obtained a total of 11,603,589 sequence reads across 751 samples (mean of 15,369 ± 54,686 reads) after quality control and further processing for visualization was performed using QIIME2 and R.

### α-Diversity and β-diversity analysis

We imported results of the QIIME2 analysis into R (R software, version 4.0.3, https://www.r-project.org) for further analysis using the standard ecological measures of microbial diversity for the number of unique taxa per sample (α-diversity) and similarity in composition between samples (β-diversity). We calculated several metrics for both α- (within-sample) diversity (observed ASV, Shannon and inverse Simpson indices) and β- (between-sample) diversity (Bray–Curtis, weighted, unweighted, and generalized UniFrac) after rarefaction and reported the metric with significant *p*-values. The various metrics measure microbial community diversity in different ways. For instance, while observed ASV qualitatively measures the microbial community richness, both Shannon and inverse Simpson indices consider both the number of taxa present as well as the abundance of each taxon in the community. In case of β-diversity, Bray–Curtis quantitatively measures of community dissimilarity without incorporating phylogenetic relationships between the taxa as is done in the various UniFrac metrics^59^. While the unweighted UniFrac only considers taxa present thereby giving weight to rare taxa, weighted UniFrac assigns weight to the dominant taxa and generalized UniFrac equally favors taxa of varying abundance^59,60^. To assess the association with α-diversity, we fitted a linear regression model (“lm” function in R “stats” package v4.1.2) and determined statistical significance using the t-statistic. Using the permutational multivariate analysis of variance (PERMANOVA), a distance-based analysis of variance method based on permutation (999 permutations, “adonis” function in the R “vegan” package 2.5–7), we tested the association between the various factors of interest (e.g., group, stage, grade, and histology of OC) and β-diversity measures after rarefying the data. We also adjusted for significantly different covariates (Table 1: batch, age, BMI, and menopausal status) and then generated ordination plots using classic multi-dimensional scaling (MDS) as implemented in R (“cmdscale” function in the R “stats” package v4.1.2). A permutation test which takes the minimum *p*-values of individual β-diversity measures as the test statistic (omnibus test), was used to combine multiple sources of association evidence provided by different β-diversity measures and an overall association *p*-value was reported (“PermanovaG” function in the R “GUniFrac” package v1.4).

### Differential abundance analysis

We completed differential analysis at the species level, filtering rare taxa prevalent at less than 10% of samples or taxa with a maximum proportion (relative abundance) less than 0.2% to reduce the number of necessary tests. We utilized the R package LinDA (linear models for differential abundance analysis), a linear regression framework for differential abundance analysis that fits linear regression models on centered log-ratio transformed data, identifies a bias term due to the transformation and compositional effect, and corrects the bias using the mode of the regression coefficient^61^. The p-values were computed based on the bias-corrected regression coefficients and the Benjamini–Hochberg procedure used to control the false discovery rate (FDR). We assessed statistical significance with FDR-adjusted p-values less than 0.10 considered.

### Covariate adjustment

We adjusted for covariates (batch, age, BMI, menopausal status, history of hypertension, stage, grade, histology and debulking status) that were differentially present between comparison groups and that showed a significant microbiome impact after a PERMANOVA analysis.

## Supplementary Information

Supplementary Figures.

Supplementary Tables.

## Data Availability

The raw sequence dataset supporting the results of this article has been publicly deposited and are available at the NCBI Sequence Read Archive (SRA), with BioProject ID PRJNA836143 (http://www.ncbi.nlm.nih.gov/bioproject/836143).
