# Supplementary material for: Diagnostic and prognostic potential of the microbiome in ovarian cancer treatment response
Source: Sci Rep. 2023 Jan 13;13:730. doi: 10.1038/s41598-023-27555-x (PMC9839674; doi:10.1038/s41598-023-27555-x)
Supplement: Supplementary file 1 — Supplementary Figures. [file 41598_2023_27555_MOESM1_ESM.docx]

Supplemental Figure 1: Genus-level microbial community composition (Abundance and relative abundance) plots of patients with or without OC and controls. **(A)** Microbial taxa abundance plots of all samples including controls. **(B)** Microbial taxa relative abundance plots of all samples including controls. **(C)** Microbial taxa abundance plots of only controls. **(D)** Microbial taxa relative abundance plots of all only controls. PCR-NTC: PCR negative control, PCR-NTC-GEO: PCR negative control with *Geobacillus*, TE-GEO: TE with *Geobacillus*. Only microbial taxa present at a minimum of 1 % relative frequency in at least one sample are shown for graphical clarity.

Supplemental Figure S2: Bacterial community α-diversity between patients with and without OC. A Wald statistical test was performed and Observed ASVs, Shannon Index and Inverse Simpson were reported. **(A)** Lower reproductive tract (cervix and vagina), **(B)** Uterus, **(C)** Fallopian tubes, **(D)** Ovaries, **(E)** Ascites/Peritoneal fluid, **(F)** Urine. Analysis was adjusted for menopause status, and BMI. *Groups are significantly different, and *p*-values are shown only for these groups.

Supplemental Figure S3: Bacterial community β-diversity between patients with and without OC. Bray-Curtis, unweighted, weighted, and generalized UniFrac distance metrics were reported. **(A)** Lower reproductive tract (cervix and vagina). **(B)** Uterus. **(C)** Fallopian tubes. **(D)** Ovaries. **(E)** Ascites/Peritoneal fluid. **(F)** Urine. Analysis was adjusted for menopause status, and BMI. *Groups are significantly different, and *p*-values are shown only for these groups.

Supplemental Figure S4: Bacterial community α-diversity between patients with and without different stages of OC. A Wald statistical test was performed and Observed ASVs, Shannon Index and Inverse Simpson were reported. **(A)** Lower reproductive tract (cervix and vagina). **(B)** Uterus. **(C)** Fallopian tubes. **(D)** Ovaries. **(E)** Ascites/Peritoneal fluid. **(F)** Urine. **(G)** Stool. Analysis was adjusted for menopause status, and BMI. *Groups are significantly different, and p-values are shown only for these groups.

Supplemental Figure S5: Bacterial community β-diversity between patients with and without different stages of OC. Bray-Curtis, unweighted, weighted, and generalized UniFrac distance metrics were reported. **(A)** Lower reproductive tract (cervix and vagina). **(B)** Uterus. **(C)** Fallopian tubes. **(D)** Ovaries. **(E)** Ascites/Peritoneal fluid. **(F)** Urine. **(G)** Stool. Analysis was adjusted for menopause status, and BMI. *Groups are significantly different, and p-values are shown only for these groups.

Supplemental Figure S6: Bacterial community α-diversity between patients with and without different grades of OC. A Wald statistical test was performed and Observed ASVs, Shannon Index and Inverse Simpson were reported. **(A)** Lower reproductive tract (cervix and vagina). **(B)** Uterus. **(C)** Fallopian tubes. **(D)** Ovaries. **(E)** Ascites/Peritoneal fluid. **(F)** Omentum. **(G)** Urine. **(H)** Stool. Analysis was adjusted for menopause status, and BMI. *Groups are significantly different, and p-values are shown only for these groups.

Supplemental Figure S7: Bacterial community β-diversity between patients with and without different grades of OC. Bray-Curtis, unweighted, weighted, and generalized UniFrac distance metrics were reported. **(A)** Lower reproductive tract (cervix and vagina). **(B)** Uterus. **(C)** Fallopian tubes. **(D)** Ovaries. **(E)** Ascites/Peritoneal fluid. **(F)** Omentum. **(G)** Urine. **(H)** Stool. Analysis was adjusted for menopause status, and BMI. *Groups are significantly different, and p-values are shown only for these groups.

Supplemental Figure S8: Bacterial community α-diversity between patients with and without different histologies of OC. A Wald statistical test was performed and Observed ASVs, Shannon Index and Inverse Simpson were reported. **(A)** Lower reproductive tract (cervix and vagina). **(B)** Uterus. **(C)** Fallopian tubes. **(D)** Ovaries. **(E)** Ascites/Peritoneal fluid. **(F)** Omentum. **(G)** Urine. **(H)** Stool. Analysis was adjusted for menopause status, and BMI. *Groups are significantly different, and p-values are shown only for these groups.

Supplemental Figure S9: Bacterial community β-diversity between patients with and without different histologies of OC. Bray-Curtis, unweighted, weighted, and generalized UniFrac distance metrics were reported. **(A)** Lower reproductive tract (cervix and vagina). **(B)** Uterus. **(C)** Fallopian tubes. **(D)** Ovaries. **(E)** Ascites/Peritoneal fluid. **(F)** Omentum. **(G)** Urine. **(H)** Stool. Analysis was adjusted for menopause status, and BMI. *Groups are significantly different, and p-values are shown only for these groups.

Supplemental Figure S10: Bacterial community α-diversity between OC patients with different tumor responses to treatment. A Wald statistical test was performed and Observed ASVs, Shannon Index and Inverse Simpson were reported. **(A)** Lower reproductive tract (cervix and vagina). **(B)** Uterus. **(C)** Fallopian tubes. **(D)** Ovaries. **(E)** Ascites/Peritoneal fluid. **(F)** Omentum. **(G)** Urine. **(H)** Stool. Analysis was adjusted for menopause status, and BMI. *Groups are significantly different, and p-values are shown only for these groups.

Supplemental Figure S11: Bacterial community β-diversity between OC patients with different tumor responses to treatment. Bray-Curtis, unweighted, weighted, and generalized UniFrac distance metrics were reported. **(A)** Lower reproductive tract (cervix and vagina). **(B)** Uterus. **(C)** Fallopian tubes. **(D)** Ovaries. **(E)** Ascites/Peritoneal fluid. **(F)** Omentum. **(G)** Urine. **(H)** Stool. Analysis was adjusted for menopause status, and BMI. *Groups are significantly different, and p-values are shown only for these groups.

Supplemental Figure S12: Bacterial community α-diversity between OC patients with different statuses two years post-diagnosis. A Wald statistical test was performed and Observed ASVs, Shannon Index and Inverse Simpson were reported. **(A)** Lower reproductive tract (cervix and vagina). **(B)** Uterus. **(C)** Fallopian tubes. **(D)** Ovaries. **(E)** Ascites/Peritoneal fluid. **(F)** Omentum. **(G)** Urine. **(H)** Stool. Analysis was adjusted for menopause status, and BMI. *Groups are significantly different, and p-values are shown only for these groups.

Supplemental Figure S13: Bacterial community β-diversity between OC patients with different statuses two years post-diagnosis. Bray-Curtis, unweighted, weighted, and generalized UniFrac distance metrics were reported. **(A)** Lower reproductive tract (cervix and vagina). **(B)** Uterus. **(C)** Fallopian tubes. **(D)** Ovaries. **(E)** Ascites/Peritoneal fluid. **(F)** Omentum. **(G)** Urine. **(H)** Stool. Analysis was adjusted for menopause status, and BMI. *Groups are significantly different, and p-values are shown only for these groups.

Supplemental Figure S14: Bacterial community α-diversity between OC patients with different statuses four years post-diagnosis. A Wald statistical test was performed and Observed ASVs, Shannon Index and Inverse Simpson were reported. **(A)** Lower reproductive tract (cervix and vagina). **(B)** Uterus. **(C)** Fallopian tubes. **(D)** Ovaries. **(E)** Ascites/Peritoneal fluid. **(F)** Omentum. **(G)** Urine. **(H)** Stool. Analysis was adjusted for menopause status, and BMI. *Groups are significantly different, and p-values are shown only for these groups.

Supplemental Figure S15: Bacterial community β-diversity between OC patients with different statuses four years post-diagnosis. Bray-Curtis, unweighted, weighted, and generalized UniFrac distance metrics were reported. **(A)** Lower reproductive tract (cervix and vagina). **(B)** Uterus. **(C)** Fallopian tubes. **(D)** Ovaries. **(E)** Ascites/Peritoneal fluid. **(F)** Omentum. **(G)** Urine. **(H)** Stool. Analysis was adjusted for menopause status, and BMI. *Groups are significantly different, and p-values are shown only for these groups.
